# Telephone-based evaluation of cognitive impairment and mood disorders in cardiac arrest survivors with good neurologic outcomes: a retrospective cohort study

**DOI:** 10.1038/s41598-023-44963-1

**Published:** 2023-10-23

**Authors:** June-sung Kim, Youn-Jung Kim, Seung Mok Ryoo, Shin Ahn, Won Young Kim

**Affiliations:** Department of Emergency Medicine, Asan Medical Center, University of Ulsan College of Medicine, 88, Olympic-ro 43-gil, Songpa-gu, Seoul, 05505 South Korea

**Keywords:** Outcomes research, Epidemiology

## Abstract

This study determined the occurrence of cognitive impairment and mood disorders in out-of-hospital cardiac arrest (OHCA) survivors with good neurologic outcomes. We performed a retrospective, cross-sectional, single-center study with a total of 97 patients. We evaluated cognitive dysfunction via the Montreal Cognitive Assessment and Alzheimer’s disease-8 mood disorders via the Patient Health Questionnaire-9 and the Hospital Anxiety and Depression Scale. We measured quality of life with the European Quality of Life 5-Dimension 5-Levels questionnaire. Cognitive impairment and mood disorders were common among patients with good neurologic recovery. There were 23 patients who experienced cognitive impairments (23.7%) and 28 who suffered from mood disorders (28.9%). Age (adjusted OR 1.07, 95% CI 1.02–1.12), mood disorders (adjusted OR 22.80, 95% CI 4.84–107.49) and hospital length of stay (adjusted OR 1.05, 95% CI 1.02–1.09) were independent risk factors for cognitive impairment. The occurrence of cognitive impairments (adjusted OR 9.94, 95% CI 2.83–35.97) and non-cardiac causes of cardiac arrest (adjusted OR 11.51, 95% CI 3.15–42.15) were risk factors for mood disorders. Quality of life was significantly lower in the OHCA survivors with each disorder than the healthy individuals. Routine screening and intervention are needed for OHCA survivors.

## Introduction

Approximately 6–11% of patients who experience an out-of-hospital cardiac arrest (OHCA) are discharged with good neurological outcomes, as defined by a Cerebral Performance Category (CPC) score ≤ 2 or Modified Rankin Scale score ≤ 3^1,2^. However, the consequences of cardiac arrest, even in cases of favorable neurological outcomes, can limit patients’ daily activities, psychological conditions, and quality of life (QoL)^3,4^. Several studies have reported that OHCA survivors may be cognitively impaired, predominantly regarding memory and executive functions, and emotional problems, including anxiety and depression, during long-term follow-up^5–7^. However, estimates of the functional status of OHCA survivors vary widely based on the assessment method used^8,9^.

As cognitive impairment and emotional distress impact OHCA survivors’ return to daily life, recent rehabilitation guidelines emphasize assessments for cognitive and emotional problems during follow-up^10,11^. However, limited evidence is available regarding standard screening tools and protocols. An extensive neuropsychological examination may be ideal for detection; however, it is time-consuming and requires patients and their caregivers to remain in the hospital. The Mini Montreal Cognitive Assessment (MoCA) and Alzheimer’s Disease 8 (AD-8) are widely used and extensively validated screening tools for examining individuals with mild cognitive impairment^12,13^. The Patient Health Questionnaire-9 (PHQ-9) and the Hospital Anxiety and Depression Scale (HADS) are commonly employed to identify comorbid depression and anxiety in numerous medical ill patients^14^.

This study evaluated cognitive impairment and mood disorders in OHCA survivors with good neurologic outcomes using previously described scoring systems via non-face-to-face examinations at the 6-month follow-up post-cardiac arrest. We also investigated the association between the occurrence of each condition and QoL.

## Methods

### Study design and participants

We conducted this retrospective, cross-sectional, single-center study at an urban, tertiary hospital in Seoul, Korea between July 2012 and June 2021. The hospital admits nearly 400 OHCA cases and 120,000 adult patients annually to the emergency department (ED). Our hospital has prospectively registered comatose survival since 2012 for patient monitoring, improving outcomes, and systemic research^15^. Only patients undergoing targeted temperature management were included in the register. We excluded pediatric patients (< 19 years old); those with trauma, terminal illnesses, or a do-not-resuscitate order; those who refused target temperature management or admission to the intensive care unit because of cost or familial support; and those who were transferred due a lack of room.

The registry included epidemiological and clinical data, such as prehospital data, ED resuscitation according to the Utstein guidelines, and postcardiac arrest syndrome care-related factors^16^. The clinical outcomes also included duration of hospital stay, survival to discharge, and neurologic outcomes. Neurologic outcomes were evaluated with Cerebral Performance Category (CPC) scores at discharge by the emergency medicine physicians on duty and 6 months later by physicians using the telephone.

We extracted previously enrolled registry patients who had favorable neurologic outcomes (i.e., CPC 1 or 2) at both discharge and the 6-month follow-up between 2012 and 2021. We thoroughly explained the study design to the patients and obtained informed consent via telephone. Patients were excluded if they refused to participate in the study or did not have the necessary language skills. This study was approved by the Institutional Review Board of the Asan Medical Center (study no. 2023–1891). All methods were performed in accordance with the relevant guidelines and regulations.

### Measures

#### Cognitive assessments: performance-based (Mini MoCA) and informant-based (AD-8) surveys

Experienced emergency physicians (K.J.S. and K.Y.J.) interviewed the participants via the telephone to screen for cognitive impairment and mood disorders. The Mini MoCA assesses verbal fluency (rating 0–4), delayed recall (rating 0–5), and orientation (rating 0–6). Excluding the visual tests, the survey has a maximum score of 15 points, with a score ≤ 11 considered as cognitive impairment^17^. The AD-8 is an 8-item questionnaire that evaluates memory, orientation, executive functioning, and interest in activities, with a total score of 8 points (i.e., yes or no answers for each question)^18^. A score of ≥ 2 demonstrates cognitive impairment. Presence of cognitive impairment was reported if at least one of the two measures fell in the impaired range.

#### Mood assessments: HADS and PHQ-9

The PHQ-9 is a self-rating questionnaire consisting of a 9-item depression module per the Diagnostic and Statistical Manual of Mental Disorders. Responders score each item from 0 to 3 based on the frequency of their experiences over the previous 2 weeks (not at all, several days, more than half the days, or nearly every day), with a maximum score of 27 points^19^. Major depression is considered if 5 or more of the 9 depressive episodes were noted at least “more than half the days” in the previous 2 weeks and if one of the symptoms is anhedonia or depressed mood. The HADS rating scale is comprised of 14 items, 7 of which are related to anxiety and 7 to depression^20^. Each item is scored on a 4-point scale from 0–3, with the final score ranging between 0–21. A cut-off ≥ 8 was used to determine depression or anxiety. Presence of a mood disorder was reported if at least one of two measures scored above the cut-off value.

#### Quality of life assessments: EQ-5D-5L and EQ-VAS

We measured QoL with the European Quality of Life 5-Dimension 5-Levels questionnaire (EQ-5D-5L) and visual analogue scale (EQ-VAS). The EQ-5D-5L is scored on a 5-point scale, with a smaller score indicating a more positive outcome, and combined to form a 5-digit score^21^. This is then converted into an EQ utility index based on set values obtained from a reference population^22^. The EQ-VAS estimates health with a range from 0 to 100, indicating the worst to best imaginable health.

We extracted demographic data, including age, sex, and comorbidities, from the registry. Arrhythmia included any underlying arrhythmia that required treatment, such as medication, an implantable cardioverter defibrillator, pacemaker, or catheter ablation, before cardiac arrest. Malignancy was defined as active cancer confirmed through histologic or cytologic data before cardiac arrest or recurrent or metastatic cancer not in complete remission from a hematologic malignancy. We extracted prehospital and ED resuscitation-related factors according to the Utstein guidelines. Presumed causes of cardiac arrest were recorded by physicians on duty and divided into two categories (i.e., cardiac and other medical causes). We collected postcardiac arrest care-related data and clinical outcomes, such as percutaneous cardiac intervention, extracorporeal membrane oxygenation, duration of intensive care unit stays, and duration of hospital stay, from electronic medical records.

### Statistical analysis

We conducted descriptive analyses to compare baseline characteristics concerning cognitive impairment and at least one mood disorder. We presented categorical variables as percentages and analyzed them using chi-square tests or Fisher’s exact tests. We represented non-normally distributed continuous variables as medians with interquartile ranges and compared them using the Mann–Whitney U tests. We determined independent risk factors for the occurrence of each condition by backward stepwise multivariate logistic regression analyses with age and statistically significant variables (*P* < 0.1) from the univariate analysis. Odds ratio (OR) and 95% confidence interval (CI) for each variable were presented. In case of cognitive impairment, age, arrest causes, diabetes, initial rhythms, mood disorders, stroke, hospital length of stay, and CPR duration were included. For mood disorders, age, arrest causes, stroke, and cognitive impairments were contained. We verified multicollinearities among variables by using the variance inflation factor before conducting adjusted logistic regression models. We performed all statistical analyses using SPSS version 21.0 (IBM Corp., Armonk, NY, USA), with *P* < 0.05 considered statistically significant.

## Results

**Figure 1:**
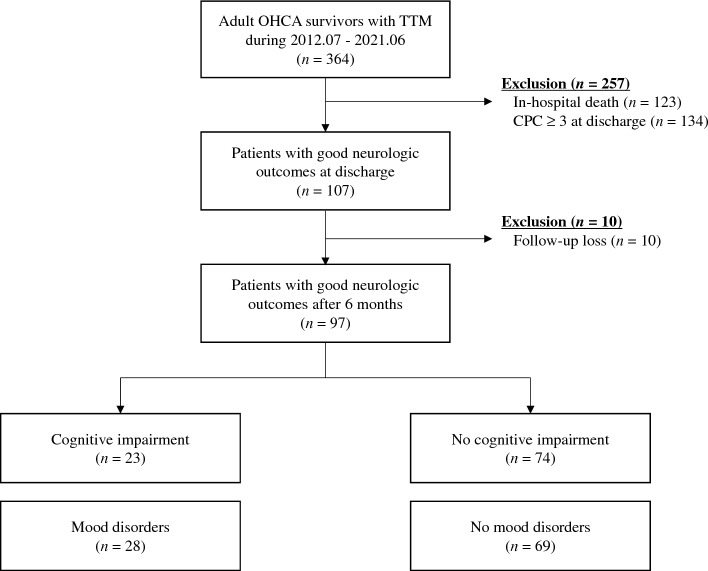
Study flowchart. There were 97 patients enrolled in the study, 23 (23.7%) of whom showed cognitive impairments and 28 (28.9%) of whom had mood disorders. OHCA: out-of-hospital cardiac arrest; TTM: targeted temperature management; CPC: cerebral performance category.

During the study period, 364 adult patients survived OHCA and underwent targeted temperature management (Fig. 1). In the final analysis, 97 patients (75.3% men; median age: 54 years [42.0–64.0]) were enrolled after excluding 267 patients due to in-hospital death (n = 123), poor neurologic outcomes at discharge (n = 134) or being unable/unwilling to undergo an examination (n = 10). Among them, 23 (23.7%) patients showed cognitive impairment and 28 (28.9%) presented with emotional disorders.

Tables 1 and 2 show the baseline characteristics of the study population according to cognitive impairment and mood disorders, respectively. Patients with cognitive impairments were older (57.0 [51.0–75.0] vs. 52.0 [40.8–61.3] years) and had more non-cardiac origin arrest (43.5% vs. 14.9%) than those without cognitive dysfunction. Moreover, a previous history of stroke (13.0% vs. 2.7%), diabetes mellitus (26.1% vs. 6.8%), and initial asystole rhythm (17.4% vs. 2.7%) were more frequent in patients with cognitive impairments than those without. The cognitive impairment group had a longer duration of hospital stay (20.0 [13.0–37.0] vs. 15.0 [12.0–20.0] days) than that of the unimpaired group. Those with mood disorders were less likely to be of the male sex (67.9% vs. 78.3%) (Table 2). A non-cardiac etiology of cardiac arrest (53.6% vs. 8.7%) was more common in patients with mood disorders. Resuscitation-related variables, including initial rhythm, duration, and postcardiac arrest syndrome treatment, did not statistically differ between the groups.

**Table 1:** Baseline characteristics of the study population according to cognitive impairments.

Variables	Total (n = 97)	No CI (n = 74)	CI (n = 23)	*P*
Men	73 (75.3)	55 (74.3)	18 (78.3)	0.70
Age	54.0 (42.0–64.0)	52.0 (40.8–61.3)	57.0 (51.0–75.0)	0.05
Arrest causes				< 0.01
Cardiac cause	76 (78.4)	63 (85.1)	13 (56.5)	
Other medical	21 (21.6)	11 (14.9)	10 (43.5)	
Past illnesses				
AMI	16 (16.5)	13 (17.6)	3 (13.0)	0.83
CHF	4 (4.1)	2 (2.7)	2 (8.7)	0.21
Stroke	5 (5.2)	2 (2.7)	3 (13.0)	0.05
HTN	31 (32.0)	24 (32.4)	7 (30.4)	0.75
DM	11 (11.3)	5 (6.8)	6 (26.1)	0.01
Malignancy	3 (3.1)	3 (4.1)	0 (0.0)	0.33
Initial rhythms				
VF or VT	20 (20.6)	14 (18.9)	6 (26.1)	0.46
PEA	11 (11.3)	9 (12.2)	2 (8.7)	0.65
Asystole	6 (6.2)	2 (2.7)	4 (17.4)	0.01
Resuscitation, min	0.0 (0.0–0.95)	0.0 (0.0–5.5)	6.0 (0.0–12.0)	0.08
Total CPR duration	14.0 (8.0–25.5)	13.0 (7.8–26.3)	16.0 (9.0–25.0)	0.41
Witnessed arrest	83 (85.6)	65 (87.8)	18 (78.3)	0.25
Bystander CPR	68 (70.1)	52 (70.3)	16 (69.6)	0.85
ED shock	22 (22.7)	15 (20.3)	7 (30.4)	0.31
Therapeutic hypothermia	87 (89.7)	65 (87.8)	22 (95.7)	0.28
MD	28 (28.9)	12 (16.2)	16 (69.6)	< 0.01
ICU LOS, days	8.0 (6.0–10.0)	8.0 (6.0–10.0)	8.0 (6.0–11.0)	0.34
Hospital LOS, days	16.0 (12.0–21.0)	15.0 (12.0–20.0)	20.0 (13.0–37.0)	0.02

The data are presented as median (interquartile range) or number (percentage). We conducted the Chi-square tests or Fisher’s exact tests for categorical variables and Mann–Whitney U tests for non-normally distributed continuous variables.

CI: cognitive impairment; AMI: acute myocardial infarction; CHF: congestive heart failure; HTN: hypertension; DM: diabetes mellitus; VF: ventricular fibrillation; VT: ventricular tachycardia; PEA: pulseless electrical activity; ED: emergency department; CPR: cardiopulmonary resuscitation; MD: mood disorders; ICU: intensive care unit; LOS: length of stay.

**Table 2:** Baseline characteristics of the study population according to mood disorders.

Variables	Total (n = 97)	No MD (n = 69)	MD (n = 28)	*P*
Men	73 (75.3)	54 (78.3)	19 (67.9)	0.28
Age	54.0 (42.0–64.0)	55.0 (44.0–63.5)	51.5 (31.0–65.8)	0.46
Arrest causes				< 0.01
Cardiac	76 (78.4)	63 (91.3)	13 (46.4)	
Other medical	21 (21.6)	6 (8.7)	15 (53.6)	
Past illnesses				
AMI	19 (19.6)	16 (23.1)	3 (10.7)	0.19
CHF	4 (4.1)	4 (5.8)	0 (0.0)	0.19
Stroke	5 (5.2)	1 (1.4)	4 (14.3)	0.01
HTN	31 (32.0)	20 (29.0)	11 (39.3)	0.32
DM	11 (11.3)	7 (10.1)	4 (14.3)	0.56
Malignancy	3 (3.1)	2 (2.9)	1 (3.6)	0.86
Initial rhythms				0.22
VF or VT	20 (20.6)	13 (18.8)	7 (25.0)	
PEA	11 (11.3)	8 (11.6)	3 (10.7)	
Asystole	6 (6.2)	2 (2.9)	4 (14.3)	
ED resuscitation time, min	0.0 (0.0–9.5)	0.0 (0.0–5.0)	3.0 (0.0–10.8)	0.21
Total CPR duration, min	14.0 (8.0–25.5)	15.0 (9.5–27.0)	12.0 (6.3–20.8)	0.14
Witnessed arrest	83 (85.6)	59 (85.5)	24 (85.7)	0.98
Bystander CPR	68 (70.1)	48 (69.6)	20 (71.5)	0.29
ED shock	22 (22.7)	15 (21.7)	7 (25.0)	0.73
ECPR	9 (9.4)	8 (11.8)	1 (3.6)	0.21
Therapeutic hypothermia	87 (89.7)	62 (89.9)	25 (89.3)	0.93
CI	23 (23.7)	7 (10.1)	16 (57.1)	< 0.01
ICU LOS, days	8.0 (6.0–10.0)	8.0 (6.0–10.0)	8.5 (7.0–11.0)	0.30
Hospital LOS, days	16.0 (12.0–21.0)	15.0 (12.0–20.5)	18.0 (12.3–25.3)	0.35

The data are presented as median (interquartile range) or number (percentage). We conducted Chi-square tests or Fisher’s exact tests for categorical variables and Mann–Whitney U tests for non-normally distributed continuous variables.

MD: mood disorders; AMI: acute myocardial infarction; CKD: chronic kidney disease; CHF: congestive heart failure; HTN: hypertension; DM: diabetes mellitus; VF: ventricular fibrillation; VT: ventricular tachycardia; PEA: pulseless electrical activity; ED: emergency department; CPR: cardiopulmonary resuscitation; ECPR: extracorporeal membrane oxygenation cardiopulmonary resuscitation; CI: cognitive impairment; ICU: intensive care unit; LOS: length of stay.

Table 3 shows the rating of the outcome measures for screening cognitive impairments and mood disorders. All subscales for the Mini MoCA test, including verbal fluency, orientation, and delayed recall, were statistically different among groups. Patients with cognitive impairments had lower scores of the Mini MoCA (10 vs. 14), and higher scores of the AD8 (4 vs. 1) than that of patients with cognitive impairments. Three patients (1 for the Mini MoCA, and 2 for the AD8) had discrepancies for two test results. Meanwhile, all patients with mood disorders had higher PHQ9 (14 vs. 2), HADS-anxiety subscale (10 vs. 2), and HADS-depression subscale (11 vs. 3) than that of patients without mood disorders.

**Table 3:** Rating of the outcome measures for screening cognitive impairments and mood disorders.

Disorders	Scoring tool	Number (%)^a^	Without disorder	With disorder	*P*
CI (n = 23)	Mini MoCA verbal fluency (range 0–4)	–	4 (3–4)	2 (2–3)	< 0.01
	Mini MoCA orientation (range 0–6)	–	6 (5–6)	4 (3–5)	< 0.01
	Mini MoCA delayed recall (range 0–5)	–	5 (4–5)	3 (2–3)	< 0.01
	Mini MoCA total (range 0–15)	22 (22.7)	14 (14–15)	10 (8–11)	< 0.01
	AD8 (range 0–8)	21 (21.6)	1 (0–1)	4 (3–5)	< 0.01
MD (n = 28)	PHQ9 (range 0–27)	28 (28.9)	2 (1–4)	14 (6–19)	< 0.01
	HADS-A (range 0–21)	21 (21.6)	2 (1–6)	10 (8–13)	< 0.01
	HADS-D (range 0–21)	15 (15.5)	3 (2–6)	11 (9–14)	< 0.01

The data are presented as median (interquartile range) or number (percentage).

CI: cognitive impairments; MD: mood disorders; Mini MoCA: Mini Montreal Cognitive assessment; AD: Alzheimer disease; PHQ: patients health questionnaire; HADS-A: hospital anxiety-depression score for anxiety subscale, HADS-D: hospital anxiety-depression score for depression subscale.

^a^Percentage was calculated by divide the number of patients those who had below (Mini MoCA) or above (AD8, PHQ0, HADS) cut-off value into total study population (n = 97).

We performed univariate and multivariate logistic regression analyses to predict the occurrence of cognitive impairment and mood disorders. Age (adjusted OR 1.07, 95% CI 1.02–1.12), the occurrence of mood disorders (adjusted OR 22.80, 95% CI 4.84–107.49), and duration of hospital stay (adjusted OR 1.05, 95% CI 1.02–1.09) were independent risk factors for cognitive impairment (Table 4). The presence of cognitive impairment (adjusted OR 9.94, 95% CI 2.83–35.97) and arrest causes (adjusted OR 11.51, 95% CI 3.15–42.15) were independent risk factors for mood disorders (Table 5).

**Table 4:** Univariate and multivariate logistic regression for predicting the occurrence of cognitive impairments.

Variables	Univariate			Multivariate		
	OR	95% CI	*P*	Adjusted OR	95% CI	*P*
Age	1.07	1.01–1.13	0.02	1.07	1.02–1.12	0.01
Arrest causes	1.67	0.87–3.19	0.12			
DM	2.45	0.28–21.52	0.42			
Initial rhythms	1.29	0.69–2.41	0.42			
Mood disorders	23.56	4.53–122.34	< 0.01	22.80	4.84–107.49	< 0.01
Stroke	0.51	0.03–9.57	0.88			
Hospital LOS	1.05	1.01–1.08	< 0.01	1.05	1.02–1.09	< 0.01
ED CPR time	0.99	0.94–1.06	0.88			

We performed backward stepwise multivariate logistic regression analyses with statistically significant variables (P < 0.1) from the univariate analysis.

OR: odds ratio; CI: confidence interval; DM: diabetes mellitus; LOS: length of stay; ED: emergency department; CPR: cardiopulmonary resuscitation.

**Table 5:** Univariate and multivariate logistic regression for predicting the occurrence of mood disorders.

Variables	Univariate			Multivariate		
	OR	95% CI	*P*	Adjusted OR	95% CI	*P*
Age	0.97	0.93–1.01	0.17			
Arrest causes	8.45	2.09–34.10	< 0.01	11.51	3.15–42.15	< 0.01
Stroke	21.34	1.26–36.39	0.03	12.87	0.89–18.71	0.06
Cognitive impairments	14.14	3.25–61.46	< 0.01	9.94	2.83–34.97	< 0.01

We performed backward stepwise multivariate logistic regression analyses with statistically significant variables (P < 0.1) from the univariate analysis.

OR: odds ratio; CI: confidence interval.

**Figure 2:**
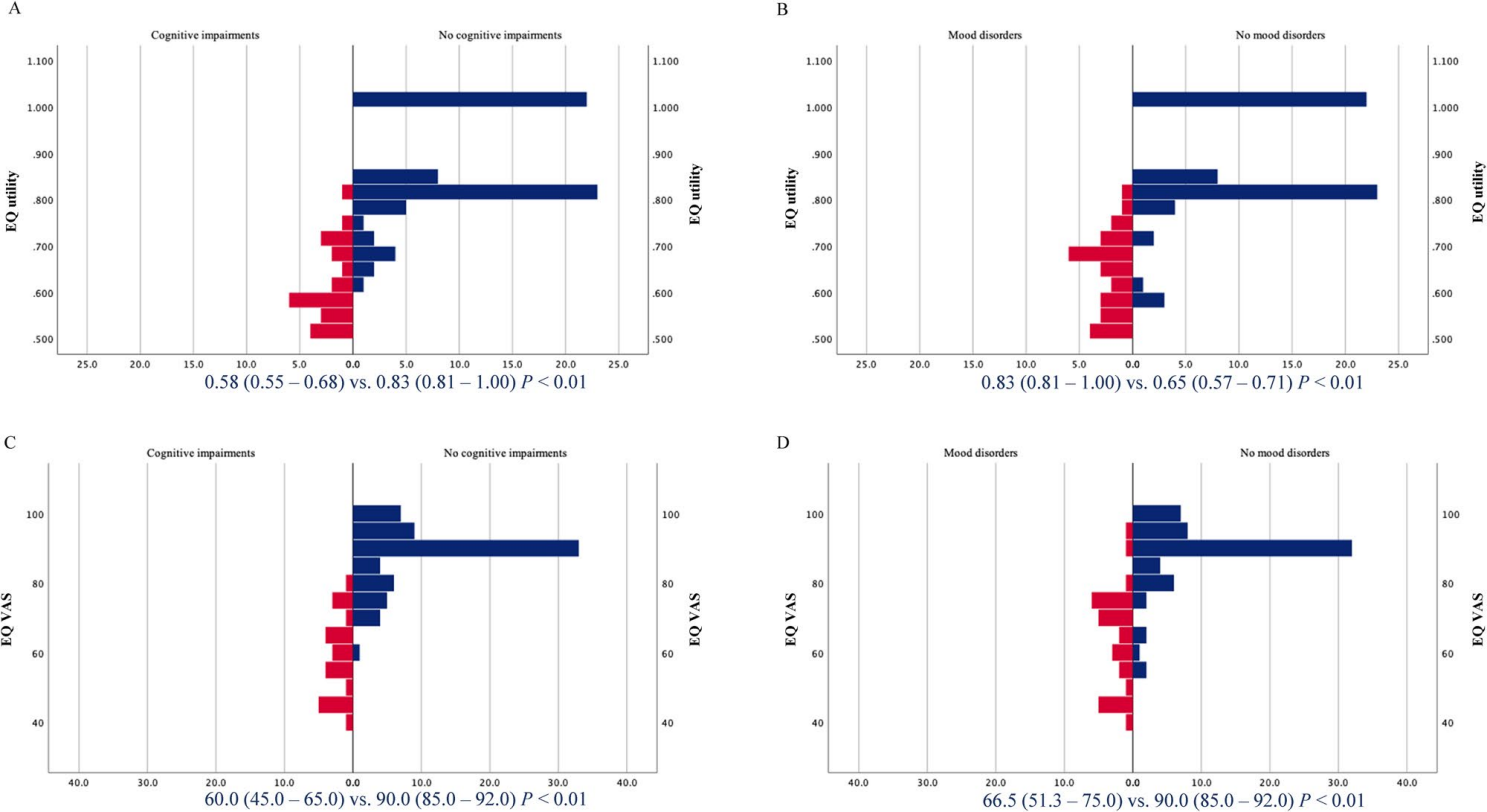
Quality of life comparisons for each impairment. A comparison of the EQ utility index of those who had cognitive impairments vs. those who did not (**A**) and those who had mood disorders vs. those who did not (**B**). A comparison of the EQ VAS score of those who had cognitive impairments vs. those who did not (**C**) and those who had mood disorders vs. those who did not (**D**). The EQ utility index and EQ-VAS showed similar trends, as those who had a disorder had significantly lower QoL than those who did not.

Figure 2 compares the QoL according to each impairment. EQ utility index and EQ-VAS showed that the patients with each disorder had significantly lower QoL than the healthy individuals.

## Discussion

In this cross-sectional study, a telephone-based evaluation demonstrated that approximately a quarter of OHCA survivors with favorable neurologic outcomes experienced cognitive impairments or mood disorders. Cognitive impairment was an independent risk factor for mood disorders, and vice versa. Furthermore, QoL was significantly lower in the group with both cognitive impairment and mood disorders than in the group with just mood disorders.

Enhanced survival rates are not always accompanied by favorable neurologic outcomes. Moreover, patients with good CPC scores still have some degree of ischemic and reperfusion brain damage due to global hypoxia^23,24^. The distribution of typically affected areas includes the medial temporal lobes, neocortex, and cerebellum, which are related to memory and executive function impairments^25^. Frontotemporal involvement was also reported in ischemic brain injury and correlates with inappropriate behaviors and social maladjustment^25^. Based on this pathophysiology, recent articles have highlighted two important issues for OHCA survivors in the acute and long-term recovery processes: decreased cognitive functions and emotional problems^26^.

Previous studies have found a prevalence of cognitive impairment and mood disorders at 18–40% among OHCA survivors^27,28^. This large variation is largely due to a lack of standardized, reliable assessments and optimal timing for screening. Most previous studies enrolled a relatively small number of patients because traditional neuropsychologic examination tools are time-consuming and require specialized personnel^24^. The rapid expansion of remotely administered tools to screen cognitive impairment and mood disorders, which are increasingly necessary in clinical settings, has far-reaching applications for patient follow-up and monitoring. In the present study, we used the Mini MoCA test and AD-8, which were developed and validated for patients with Alzheimer’s disease. The occurrence of cognitive impairment (23.7%) and emotional disorders (28.9%) 6 months after a cardiac arrest is consistent with previous studies^4,5,29^. Based on HADS scale, the prevalence of anxiety and depression among OHCA survivors are previously reported to be about 20–30%^28^. Even though the screening tools that we used were not validated in patients with OHCA, the telephone versions of Mini MoCA and AD-8 for cognitive impairment and PHQ-9 and HADS for mood disorders might be feasible OHCA screening tools for long-term follow-up^30^. However, with regard to cognitive test, AD-8 could yield too many false positives when compared to a performance-based tool such as the Mini MoCA and should be interpreted cautiously.

Although a substantial amount of cognitive improvement occurs in the first 3 months after a cardiac arrest, health-related QoL can only be adequately assessed when the patient interacts in a social environment^31,32^. Research is warranted to confirm if serial evaluation of cognitive impairments and mood disorders starting 3 months post-cardiac arrest will be more sensitive^31^. Sandroni et al. found that women are less likely than men to achieve a good QoL after cardiac arrest^33^. Furthermore, a recent telephone-based survey with 2300 people with OHCA reported that women showed poorer functional outcomes and QoL after OHCA than men^34^. They postulated that treating OHCA and withdrawing life-sustaining therapy following OHCA might differ by sex, leading to different outcomes. However, other studies from the Denmark and Netherlands did not find any sex disparities^35,36^. Our results also showed no sex differences in the development of cognitive impairment and emotional disorders. This discrepancy might be due to the heterogenous nature, population, or small sample size of our study. Furthermore, other hidden confounders such as family support, marital status, and socioeconomic status, could be contributing to the results.

In the logistic regression analysis, cognitive impairment and mood disorders were independent risk factors for one another. Previous neuropsychological studies revealed that cognition and emotion are often interrelated^37^, and among the various domains of cognitive function, memory and recall are largely influenced by the patient’s emotional state^38^. Therefore, cognitive dysfunction and mood disorders may share symptoms, making it difficult to distinguish between them. Our study also found that most of the patients who suffered cognitive impairment simultaneously experienced psychological problems. Moreover, patients with cognitive dysfunction may be more likely to develop depressive episodes than patients without it. Similarly, depression might be associated with an increased risk of cognitive disturbances. However, because of the present study’s retrospective design, we could not confirm the causality of each condition.

Our study had several limitations. First, this was the first investigation to reveal cognitive impairment and mood disorders among survivors of OHCA in South Korea with a relatively small sample size. However, its results cannot be generalized to other environments as it was conducted in a single, urban hospital. Second, we did not have the subjects’ baseline cognitive function and mood status before OHCA. We assumed that all impairments were attributable to OHCA. Third, hidden confounders, such as education, finances, and familiar support, could affect cognitive dysfunction and mood disorder occurrence. Fourth, rating scales such as Mini MoCA, AD-8, and HADS were not fully validated to recognize cognitive impairments and mood disorders in OHCA patients. Because of the COVID pandemic, it was difficult to examine patients face-to-face. Moreover, these scores might have different sensitivity and specificity for recognizing cognitive impairments and mood disorders because they had partially different constructs. Although the cut-off values of were adapted from other populations, our study results implied that the performed scores could be good screening tools for OHCA survivors. However, this must be validated with a prospective study with a large cohort. Fifth, we could include only patients who underwent TTM, which could lead to selection bias.

## Conclusions

A telephone-based evaluation of OHCA survivors with favorable neurologic outcomes 6 months post-cardiac arrest demonstrated that 23.7% had cognitive impairment and 28.9% had emotional disorders. Furthermore, both of the conditions were associated with a low QoL, necessitating appropriate screening and active intervention. A telephone-based evaluation is a convenient and simple tool to assess postcardiac arrest survivors in clinical practice.

## Data Availability

The data from this study can be obtained from the corresponding author upon reasonable request.
